# QuPath Edu and OpenMicroanatomy: Open‐source virtual microscopy tools for medical education

**DOI:** 10.1111/joa.14172

**Published:** 2024-11-18

**Authors:** Aaron Yli‐Hallila, Peter Bankhead, Mark J. Arends, Petri Lehenkari, Sanna Palosaari

**Affiliations:** ^1^ Translational Medicine Research Unit, Medical Faculty University of Oulu Oulu Finland; ^2^ Centre for Genomic and Experimental Medicine, Institute of Genetics and Cancer University of Edinburgh Edinburgh UK; ^3^ Edinburgh Pathology and CRUK Scotland Centre, Institute of Genetics and Cancer University of Edinburgh Edinburgh UK; ^4^ Medical Research Center Oulu University of Oulu and Oulu University Hospital Oulu Finland

**Keywords:** histology, histopathology, image analysis, medical education, microanatomy, open‐source software, virtual microscopy

## Abstract

Virtual microscopy is becoming increasingly common in both medical education and routine clinical practice. Virtual microscopy software is typically designed either for (1) training students in anatomy, histology, and histopathology, or (2) quantitative analysis—but not both simultaneously. QuPath is one of the most widely used software applications for histopathology image analysis in research and provides a comprehensive set of computational tools to evaluate histology slides. We have enhanced QuPath by developing a new extension, QuPath Edu, which adapts the software to function as an intuitive microanatomy learning environment. Additionally, we have created an entirely new, complementary software platform called OpenMicroanatomy, which provides an alternative way to access QuPath Edu teaching content through a web interface. These tools have been used in teaching of first year medical and dentistry students at the University of Oulu Medical Faculty, and we conducted a user survey for the Class of 2023 to assess the usability and student experience. In general, the introduced annotation and quiz features were appreciated by the students and the system usability of OpenMicroanatomy was considered excellent (SUS score 84.8). Together, these freely available tools enable teachers to develop and deploy innovative training material for anatomy, histopathology, quantitative analysis, and artificial intelligence in a wide range of contexts. This unique combination can provide the next generation of students with essential multidisciplinary skills.

## Graphical abstract

QuPath Edu and OpenMicroanatomy equip students for the future of medicine.

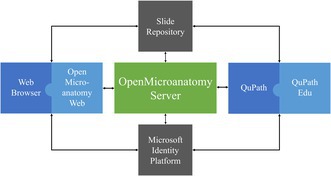

## 1 INTRODUCTION

Virtual microscopy (VM) is enabled by a combination of conventional sample preparation, whole‐slide imaging (WSI), and computing. This integration has profoundly impacted our approach to microscopy, transforming methodologies in educational, research and clinical pathology settings (reviewed in Hassell et al. (2022); Hosseini et al. (2024)). Currently, medical schools are increasingly incorporating WSI and VM into histology and histopathology curricula. VM is replacing regular optical microscopy (McBride and Drake, 2018): a process that has recently accelerated due to COVID‐19 (Maity et al., 2023). Moreover, advanced artificial intelligence (AI) technologies are being integrated into image analysis within the medical field, both for pathology and radiology (reviewed in Levenson et al. (2024)). This ongoing technological revolution is reshaping the landscape, prompting medical education to adapt and equip future medical professionals to meet these emerging challenges. Multiple studies have shown that medical students not only prefer virtual over optical microscopy, but they also perform better using VM (reviewed in Kuo and Leo (2018); Francis et al. (2023); Kumar et al. (2006); Kumar et al. (2004)). The technological interface of human–microscope is changed to human–computer, and an increasing number of open‐ and closed‐source VM and image analysis tools are available (Guerrero et al., 2022). VM has already established its presence in education and is steadily gaining momentum in routine clinical diagnostic settings (Hanna et al., 2022). In undergraduate education, the focus is on cumulative learning, and the content should support further studies in pathology and clinical sciences. However, it is both feasible and beneficial to provide selected students with additional materials on image analysis and techniques applicable to histology‐based research. As learning algorithms continue to advance, machine analysis coupled with VM has the potential to become standard practice in clinical work (Försch et al., 2021; Rodriguez et al., 2022). In clinical settings, the accuracy of the diagnosis through VM improves with the increasing experience in using VM tools (Ordi et al., 2015). Therefore, it is advisable to familiarize medical students with versatile VM tools for them to be capable of adapting to the evolving demands of future expertise and skills.

The advantages of VM in comparison with optical microscopy in teaching have been shown in the settings of medical education. Both medical and dental students demonstrate improved performance when utilizing VM, primarily attributed to the ease of use, collaborative capabilities among students, and the encouragement of self‐directed study (Kuo & Leo, 2018). As a platform VM facilitates group discussions, thereby training the students' working life skills such as consultation and learning through collaboration. The learning outcomes are further influenced by the teacher's ability to annotate slides, providing access to slides beyond the traditional classroom setting, and leading to higher satisfaction among educators (Vatchala Rani et al., 2021). Digital platforms and materials also enable self‐directed learning (Chimmalgi & Hortsch, 2022) and VM also benefits from the durability of digital slides, as they do not fade or incur damage. However, certain drawbacks revolve around technical challenges when accessing the slides and some individual studies have suggested less interaction and impaired social connections using VM (reviewed in Maity et al. (2023); Wilson et al. (2016)).

At the University of Oulu Medical Faculty, a combination of optical and VM is used to teach histology to first year medical and dentistry students. In 2018, our VM software reached its end of life and alternatives were sought. Notably, most VM software used in education (Maity et al., 2023) is commercial software designed for pathologists or closed‐source software, offering limited adaptability to our specific educational goals. In response to this, we turned to QuPath (Bankhead et al., 2017) (https://qupath.github.io/), a free and open‐source desktop application widely utilized in digital pathology research for bioimage analysis, which now has been downloaded over 500,000 times and cited in over 3000 publications (QuPath Developers, 2023). Despite being primarily designed for image analysis, QuPath's open architecture and versatility allow it to be extended for other purposes.

This article details the adaptation of QuPath for educational purposes through the creation of QuPath Edu—an open‐source extension for QuPath—and complementary software named OpenMicroanatomy, designed for enhanced accessibility and versatility. Development of QuPath Edu and OpenMicroanatomy has been ongoing at the University of Oulu since 2018, with support of the QuPath developers. Here, we describe the development and use of QuPath Edu and OpenMicroanatomy from both the teachers' and students' perspectives, presenting insights from a student survey, examples of basic histology teaching and advanced tissue analysis education, together with a succinct overview of the technical implementation of QuPath Edu and OpenMicroanatomy. For a more comprehensive understanding of the technical aspects, additional details are provided in the supplementary material.

### 1.1 Description

#### 1.1.1 QuPath Edu and OpenMicroanatomy: A complete learning environment for microanatomy education

**FIGURE 1:**
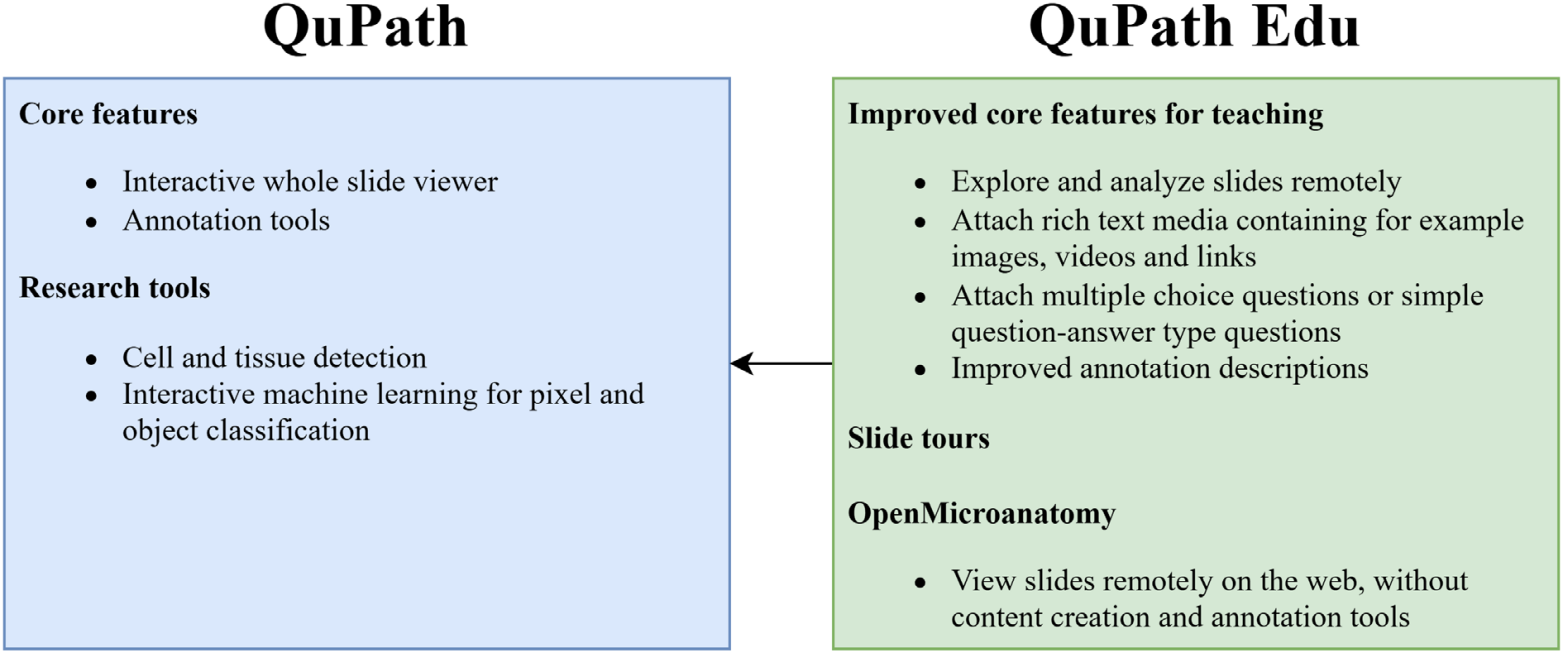
QuPath features and how they were improved for teaching with QuPath Edu. All the features improved upon QuPath Edu are also available with OpenMicroanatomy on the web.

QuPath is a freely available desktop application compatible with Windows, Mac, and Linux, which offers extensive features to view and analyze whole‐slide images. These include an interactive user interface (UI), image exploration with zooming capabilities, robust annotation and visualization tools, and research‐oriented features such as cell and tissue detection, and machine learning. QuPath Edu is an extension that is compatible with QuPath versions from 0.3.0 to 0.5.1 (the latest version at the time of writing). QuPath Edu transforms QuPath from being primarily an image analysis tool into becoming an intuitive microanatomy learning environment. QuPath Edu adds numerous enhancements, including new annotation types, the ability to embed rich text media to slides, support for slides hosted remotely, and a novel feature named “slide tours” (Figure 1 **)**. Because it is an extension, QuPath Edu ensures that users can leverage not only the current functionality, but also any new features and enhancements introduced in future versions of QuPath. This seamless integration allows users to benefit from an expansive toolset while staying abreast of advancements in QuPath's development.

**FIGURE 2:**
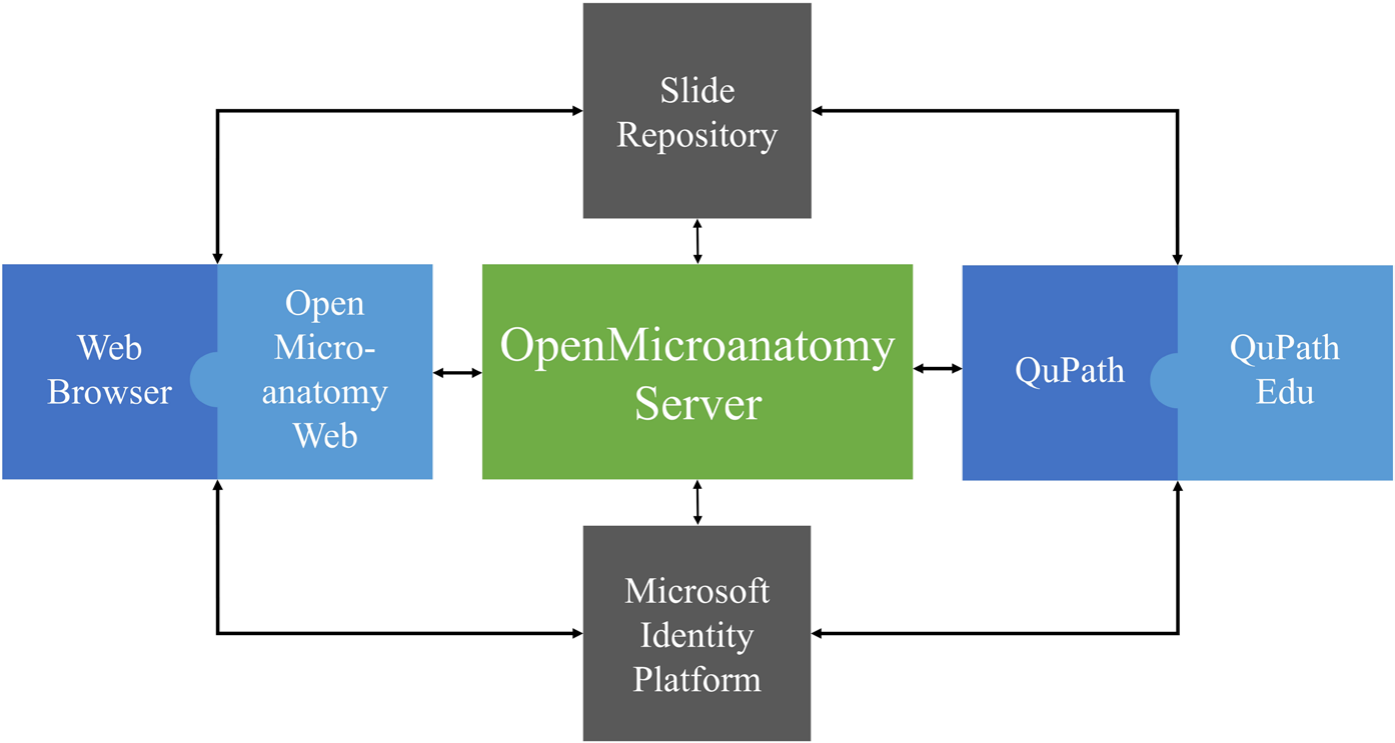
A diagram illustrating the various components of QuPath Edu and OpenMicroanatomy and how they interact with each other. The gray components represent services provided by a third party. QuPath Edu and OpenMicroanatomy Web establish communication with the OpenMicroanatomy Server, particularly when tasks such as downloading course materials or authentication are required. The Microsoft Identity Platform is an optional component, utilized only when users authenticate using Microsoft credentials. Additionally, the diagram includes a Slide Repository, representing a third‐party repository for slides (such as OMERO, Microsoft Azure, Amazon S3 or CSC Allas). The OpenMicroanatomy Server uploads slides to the Slide Repository, and end‐users can access these slides through either OpenMicroanatomy Web or QuPath Edu. It is important to note that the Slide Repository is optional, as the OpenMicroanatomy Server can independently function as a Slide Repository as well.

OpenMicroanatomy consists of two components: a server and a web client. QuPath is primarily designed for offline use with all data saved locally. In a teaching context, it is usually preferable to manage the training content centrally, and therefore, QuPath Edu can be used to develop and access training material stored online. This is achieved through communicating with the OpenMicroanatomy Server, which facilitates the sharing and synchronization of QuPath data files—making these files accessible from anywhere via the Internet. OpenMicroanatomy Web serves as a lightweight web‐based alternative to QuPath Edu, offering a more streamlined set of features. While it has limited functionalities compared to QuPath Edu, OpenMicroanatomy Web is designed to work seamlessly on web browsers and smartphones without the need for installation (Figure 2). The primary difference in features is the absence of any analysis or annotating tools that are present in QuPath (feature comparison in Table 1.). OpenMicroanatomy Web and QuPath Edu are inherently compatible with each other (Figure 2). This means that any materials created using QuPath Edu can be accessed and used on OpenMicroanatomy Web. The result is that users have the flexibility to access course materials using either the QuPath desktop application with QuPath Edu extension installed, or any modern web browser through OpenMicroanatomy Web. While OpenMicroanatomy Web is the preferred choice for learning the fundamentals of microanatomy, QuPath Edu is targeted for teachers and advanced users, particularly those interested in learning concepts related to image analysis and artificial intelligence.

**TABLE 1:** Feature comparison between QuPath Edu and OpenMicroanatomy.

Feature	QuPath	QuPath Edu	OpenMicroanatomy
Create projects	X	X	–
Analysis tools	X	X	–
View slides	X	X	–
Create content	X	X	–
Administrative tasks	–	X	–
Create workspaces	–	X	–
Improved annotations	–	X	X
Embedded rich media	–	X	X
Slide tours	–	X	X
Authentication	–	X	X
Requires installing a desktop application	X	X	–
Compatible with web browser, including smart phones	–	–	X

*Note*: X, supported; −, not supported.

**FIGURE 3:**
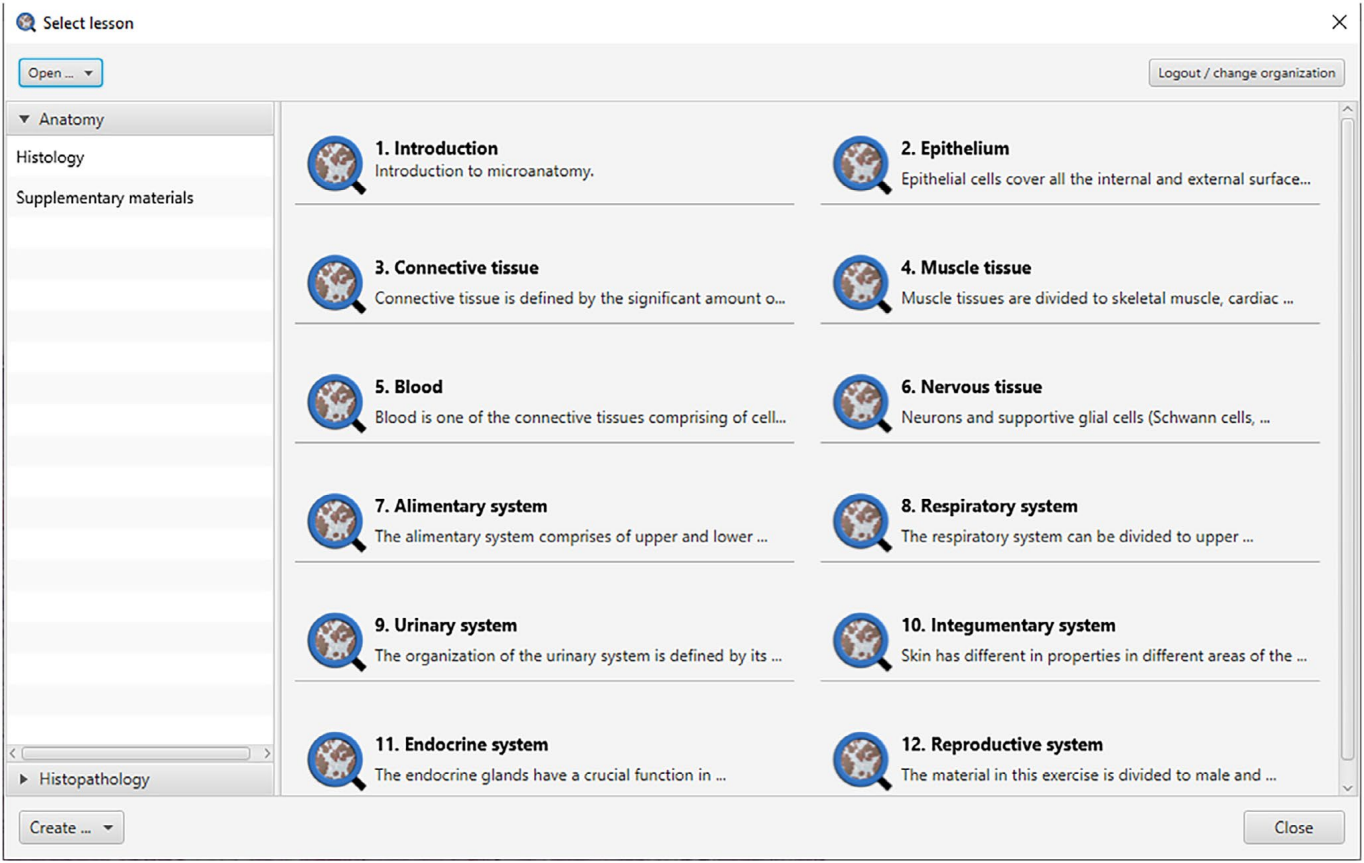
Example screenshot from the new lesson selection screen of QuPath Edu. The screenshot is of an example organization which has two workspaces: Anatomy and Histopathology. The Anatomy workspace is currently selected, and it has two courses: Histology and Supplementary Material. The Histology course is selected, and the 12 lessons belonging to the course are displayed.

QuPath Edu is designed to foster collaboration among organizations, enabling the sharing of educational materials. Whether working in parallel or independently, this collaboration occurs seamlessly on a single server (Data S1). The collaboration can be based on shared image libraries or workspaces. Under the QuPath Edu hierarchy, each entity is designated as an organization. Within each organization, there can be one or more workspaces, which represent distinct units (e.g., university departments). Each workspace encompasses courses, and these courses, in turn, house one or more lessons (Figure 3). Lessons serve as the fundamental building block of QuPath Edu, encompassing slides specific to a particular lesson and integrating all the enhancements introduced by QuPath Edu. Importantly, lessons are constructed atop QuPath's existing “project” concept, which makes it easy for users to switch between QuPath's core features and those of the Edu extension.

**FIGURE 4:**
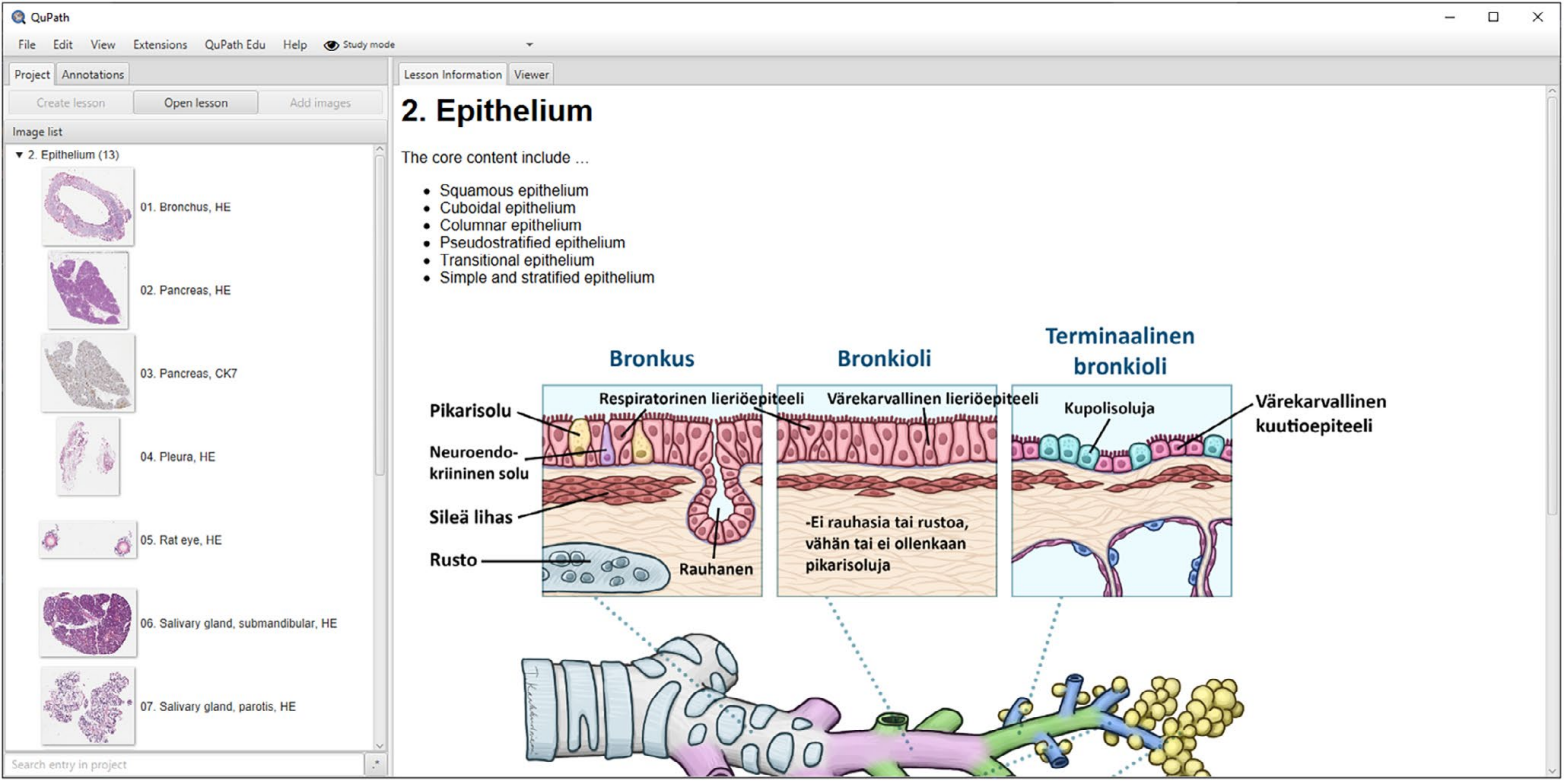
Example screenshot of QuPath Edu displaying the minimal user interface when using the “Study mode” and the new lesson information pane displaying an embedded image.

The first public versions (v. 1.0.0) of QuPath Edu and OpenMicroanatomy were released in August 2023 (https://openmicroanatomy.github.io/). Source code for QuPath Edu extension and OpenMicroanatomy is licensed under the GNU General Public License v3.0 and is available at https://github.com/openmicroanatomy. Documentation for teachers, students, and IT administrators is available at https://openmicroanatomy.github.io/docs.

#### 1.1.2 QuPath Edu and OpenMicroanatomy in basic education

**FIGURE 5:**
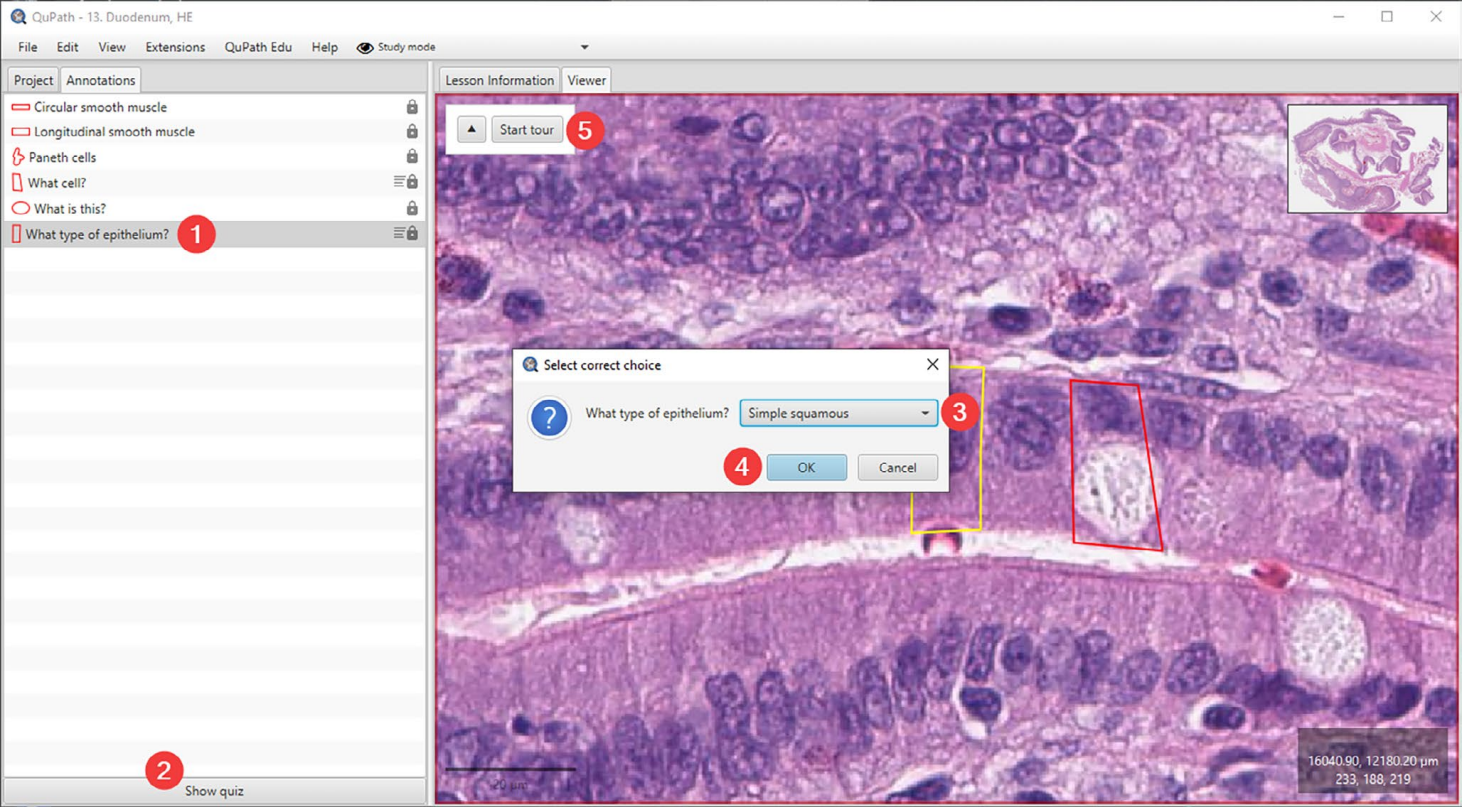
Example of a quiz annotation in QuPath Edu. Annotation is first selected (1), the quiz menu opens by pressing “Show quiz” (2) and the student can select the answer from the dropdown menu (3). Answer is submitted by pressing “OK” (4). The prompted new dialog shows the right answer, and any additional information or hints provided by the teacher. The operation is identical with OpenMicroanatomy Web. Additionally, the slide tour (see Figure 7.) can be activated by pressing “Start tour” (5).

**FIGURE 6:**
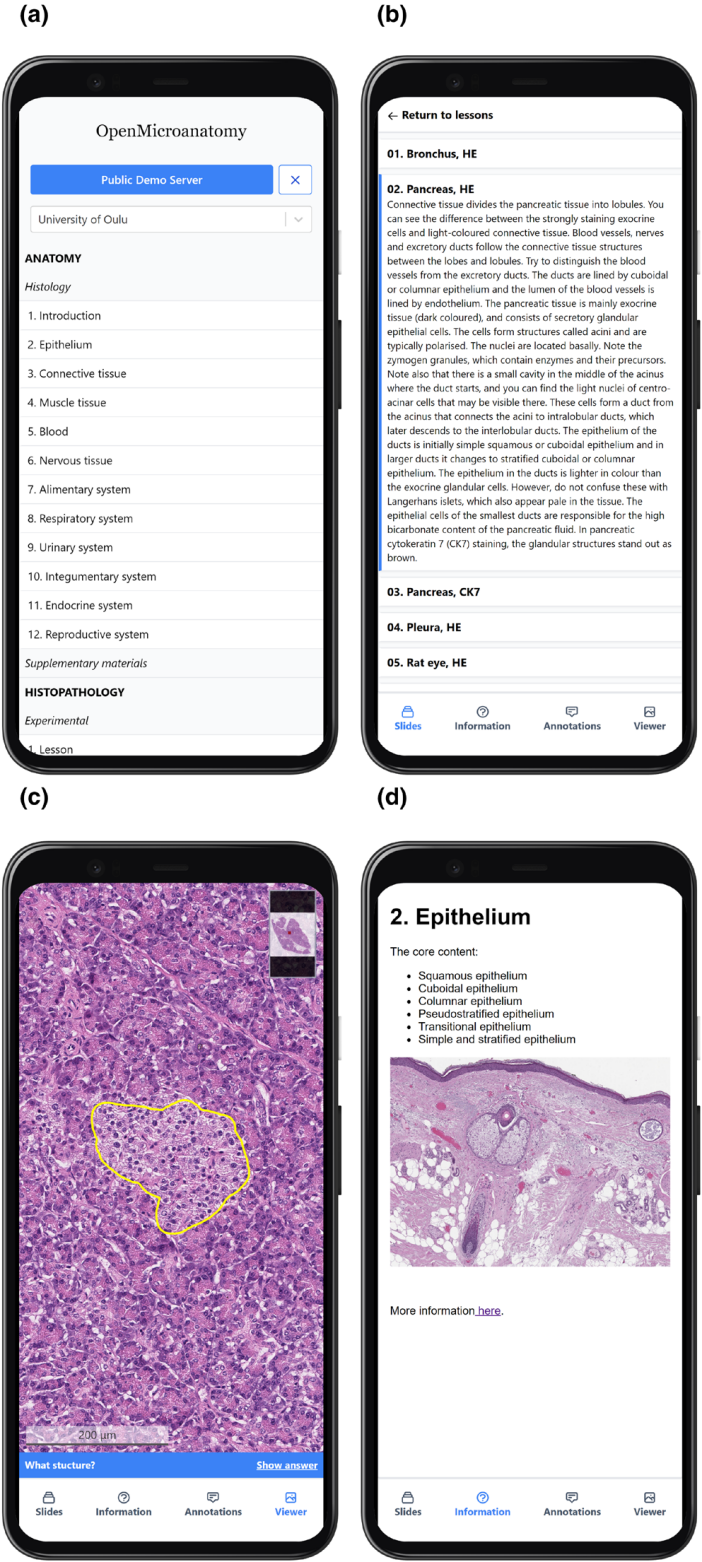
OpenMicroanatomy Web on a smartphone. (a) depicts the lesson selection screen, (b) displays the slide selection screen, (c) exhibits the image view and (d) the lesson information screen. In the image view, a slide of the pancreas is open, with a highlighted “What structure?” annotation. All annotations are accessible in the “Annotations” tab located at the bottom of the screen.

In basic education, QuPath Edu's lesson information view facilitates learning by supporting the embedding of rich text, which includes text formatting, links, images, and video. This enables comprehensive background information to be incorporated, such as introductions to the subject matter, or links to electronic textbooks or other learning resources. The lesson information view opens by default, and accessing the slides from the sidebar displays the chosen slide in the viewer (Figures 3, 4, 5, 6). Opening a slide also reveals the slide description in the sidebar and displays the slide's annotations in the annotations tab.

Slides within QuPath Edu can be annotated using various features, including slide tours, explanations, questions with hidden answers, or multiple‐choice questions. QuPath has built‐in tools for creating annotations in various sizes and shapes, and these can be assigned a name, color, and a brief description. QuPath Edu enhances the annotations by adding the possibility to include annotations in the form of questions and answers. These questions can take various formats, including open‐ended questions without specific answers, simple focused questions with a more specific answer, or multiple‐choice questions (Figure 5). Depending on the question format, either *show answer* or *show quiz* tab will appear at the bottom of the sidebar when entering the question.

**FIGURE 7:**
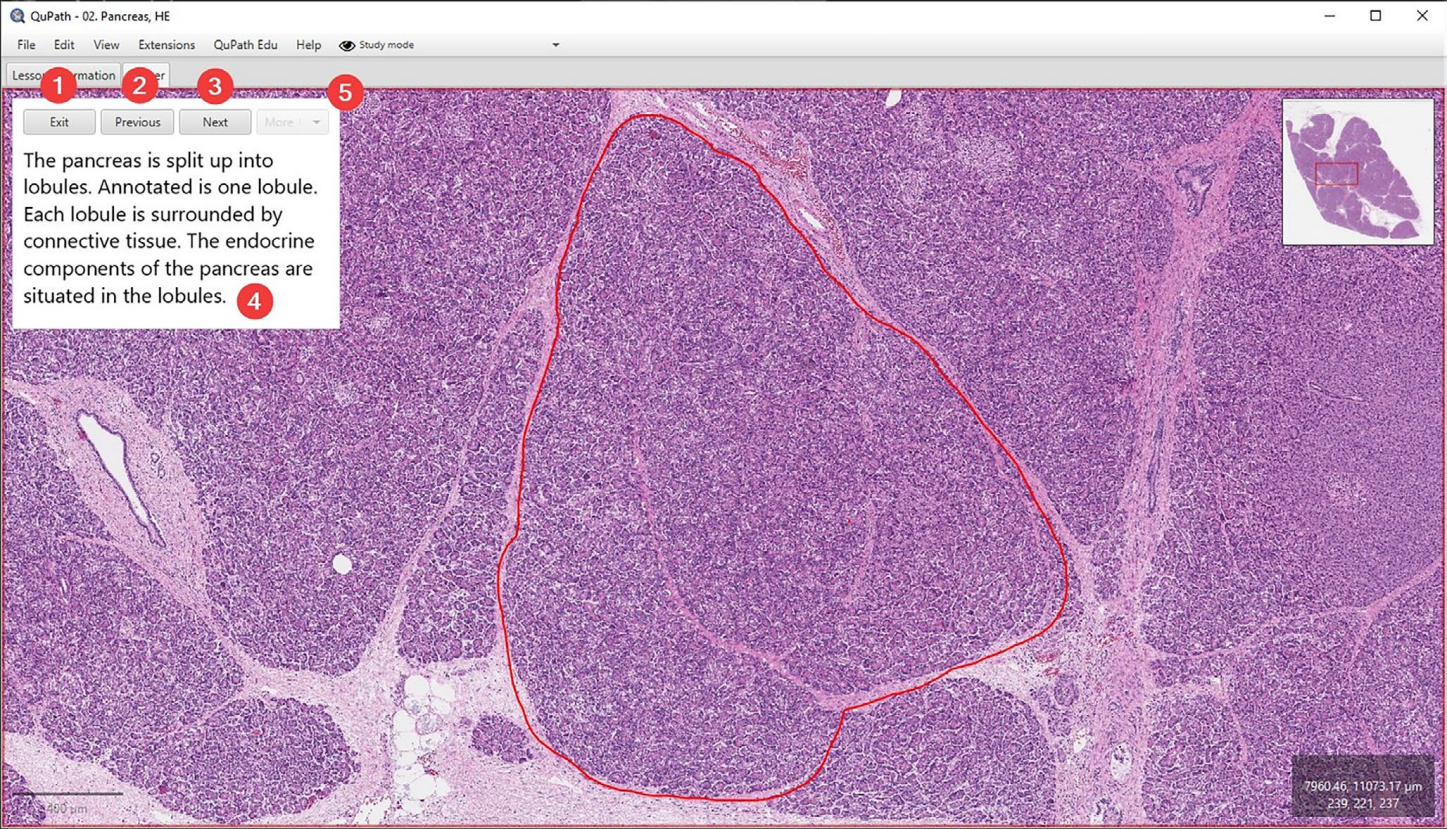
Example of a slide tour in QuPath Edu. Notice the annotation pane is hidden during a tour. The tour can be stopped by pressing “Exit” (1). The frames of the tour can be switched by pressing “Previous” and “Next” (2 and 3). A short description (4) is attached to each frame. The tools for teachers to create new frames can be found in the dropdown menu “More…” (5) which is only available in the Editing mode.

**FIGURE 8:**
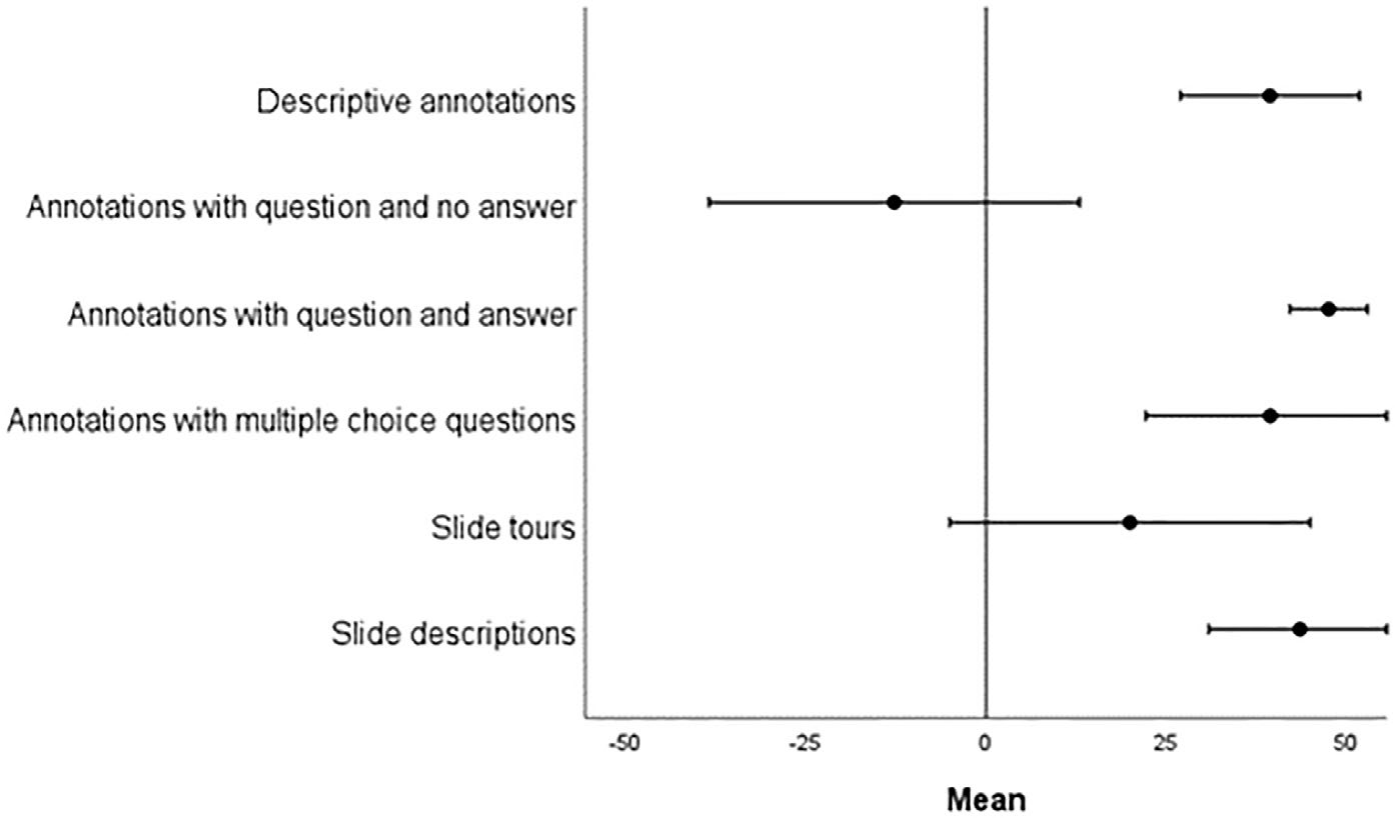
How students (*n* = 75) perceived new features of QuPath Edu and OpenMicroanatomy. Values on the continuous scale range from −50 (not useful) to 0 (neutral) and 50 (very useful). Mean values and 95% confidence interval are shown.

“Slide tours” are added as a novel feature that enables the creation of a guided tour for a slide. This allows the teacher to introduce the tissue to the student systematically by navigating through regions of interest on the slide one by one. Annotations and short descriptions can be attached to each view during this process (Figure 7). The slide tour is visible on the *Annotations* tab, allowing students to use it for independent study. Within the slide tour, users can freely navigate between views, while exploring of the image at different magnifications. The underlying philosophy behind slide tours is to introduce students to a new slide gradually, bit‐by‐bit, and not to overwhelm them with a lot of new information all at once.

#### 1.1.3 QuPath Edu in advanced education

QuPath Edu allows remote access, streamlines the sharing of training material (images), and the lesson information view serves as a platform for delivering instructions effectively. Since QuPath Edu maintains all the analysis features of QuPath, it is an excellent tool for teaching histopathology image analysis. QuPath's analysis features range from basic cell counting to advanced machine learning. QuPath Edu has three modes, which the user can switch between from the toolbar. The three modes are as follows: Study mode, Analysis mode, and Editing mode. In the basic Study mode, QuPath Edu allows slides and annotations to be viewed only. Editing mode allows teachers to create and edit content. In the Analysis mode, QuPath Edu provides access to all the analysis features inherent in QuPath, including the ability to use QuPath scripts.

QuPath Edu can be utilized in either guest mode or as signed in. The guest mode is the default setting and changes made by the user are not saved. The sign‐in option in QuPath Edu empowers students to create their own personalized copies of lessons. This feature allows students to save any changes made during the analysis work in their personal copies. Consequently, students can continue and modify their analysis work during training sessions, fostering a more personalized and interactive learning experience. By default, the copies of lessons created by students are saved for a period of 12 months. After this timeframe, these copies will be automatically removed from the server. This time limit is designed to manage storage resources and ensures that the server remains uncluttered with outdated copies of lessons.

## 2 METHODS

### 2.1 Evaluating OpenMicroanatomy user experience

The University of Oulu is among the five universities in Finland responsible for educating medical practitioners. Annually, the medical faculty enrolls 150 medical students and 50 dentistry students. These students commence their studies with anatomy, and the microanatomy course is incorporated into the first semester. The microanatomy course comprises 30 h of on‐site exercises divided into 14 topics. Prior and during the exercises, the students use the annotated virtual tissue specimens available in QuPath Edu and/or OpenMicroanatomy on their own devices. The annotations are either explanations, questions, or quizzes on specific structures. Students leverage these annotations not only for preparation before the exercises but also as a foundational basis for discussion during the practical sessions. In the microanatomy exercises, students also have the opportunity to view optical microscopy specimens. Each exercise concludes with an examination in Moodle environment, where students are assessed on a pass or fail basis. The cumulative number of successfully passed examinations determines the final grade for the course.

In the spring of 2023, we conducted an anonymous survey among the Class of 2022, comprising 200 students. The primary goal was to gain insights into students' preferences regarding the features of QuPath Edu and OpenMicroanatomy. The questionnaire consisted of questions about the system usability and the features implemented in QuPath Edu and OpenMicroanatomy. This survey aimed to gather valuable feedback to enhance and tailor these educational tools to better meet the needs and preferences of the student population. All respondents provided an informed consent for using their answers in this study. The survey received 75 responses (a 37.5% response rate), with 59 (79%) responses from medical students and 16 (21%) from dentistry students. The details of the survey are presented in the Data S2.

## 3 RESULTS

### 3.1 Students' experiences based on the user survey

The usability of OpenMicroanatomy Web was perceived as excellent by students. The system usability score (SUS) reached 84.8, corresponding to the 96–100 percentile (top 4%) based on the Sauro‐Lewis grading (Sauro & Lewis, 2016). In terms of preferences, a significant majority (76%, *n* = 57) favored the web‐based viewer, OpenMicroanatomy Web. A smaller percentage, 12% (*n* = 9), expressed a preference for the desktop application (QuPath Edu), while an additional 12% (*n* = 9) did not have a particular preference.

The assessment of the usefulness of the introduced features was conducted using a continuous scale, with values ranging from −50 (indicating not useful) to +50 (indicating very useful). The introduction of new features in QuPath Edu and OpenMicroanatomy was generally well‐received by students, with positive feedback regarding their benefits for learning histology. However, it is worth noting that annotations featuring open‐ended questions without designated answers received a less favorable response (Figure 8). Specifically, slide descriptions were highly regarded and received a positive score of 43.5 ± 12.5, indicating their value in enhancing the learning experience. Annotations accompanied by multiple‐choice questions were also considered beneficial, scoring 39.5 ± 17.2. Annotations featuring both questions and answers received the highest rating at 47.5 ± 5.3, underscoring their significant contribution to the learning process. In contrast, annotations presenting open‐ended questions without designated answers were perceived as being of limited usefulness, with a negative score of −12.7 ± 25.6. Descriptive annotations without questions were considered to have considerable value, with a score of 39.5 ± 12.3. Slide tours, while still beneficial, received a slightly lower score of 20 ± 24.8 in this evaluation. The low score of the slide tours could be related to the low number of slide tours in the course material making them somewhat unfamiliar to the students, since slide tours were implemented only in the final lesson involving two slides. The majority of students (86.7%, *n* = 65) expressed that concealing annotation answers initially had a more positive impact on their learning compared to situations where the answers were constantly visible (8.0%, n = 6). In the optional open answers, this was further emphasized as the students considered the questions with concealed answers the best feature and described the questions without answers as frustrating. A smaller portion of the students (5.3%, *n* = 4) felt indifferent toward this aspect. Most responders (77%, *n* = 58) felt they could potentially use QuPath as a research tool in the future.

## 4 DISCUSSION

The rapid advancement of histopathology and image analysis technologies will challenge both clinicians and teachers to adapt to change. As high‐throughput WSI become routine practice, we can expect computational tools that aid image evaluation to become increasingly common in clinical practice (Zarella & Alvarez, 2022). The integration of AI tools with VM has the potential to assist pathologists in tasks such as quantitative image analysis, pre‐screening, and highlighting potential areas of interest for further examination. This also changes the role of medical doctors and the skills that they need in their work. Understanding the basic concepts behind the algorithms provides the skills to interpret image analysis data and discern the analysis errors (reviewed in Bankhead (2022)). Because of this paradigm shift, medical education must change in response. The QuPath Edu platform responds to this need by providing both a tool for basic histology education and image analysis training.

### 4.1 The current benefits of QuPath Edu and OpenMicroanatomy

From the teacher's point of view, QuPath Edu in conjunction with QuPath and OpenMicroanatomy creates a versatile platform that is compatible with a range of image file formats (Data S1) and provides excellent possibilities for sharing, building, and modifying materials, and collaborating between institutions. QuPath Edu has also been well received by the students since its introduction in the microanatomy exercises, showing the well‐established advantages of VM such as ease of use, focused images, and flexible access (reviewed in Saco et al. (2016)). While the QuPath Edu extension is required to create new content and includes a viewer for that content, most students expressed a preference to view material through OpenMicroanatomy Web. This preference may be attributed to the fact that it is accessible via a familiar web browser, requiring no installation step, and having a more streamlined user interface with fewer options. Nevertheless, a minority of users preferred to use QuPath—and this is the required option for viewing training offline or transitioning into the use of image analysis and AI. Both viewing options are therefore complementary, and users benefit from being able to select the one that best meets their needs.

One of QuPath Edu's benefits is the flexibility of its annotation options, which allow for gradual introduction of the material to students without overwhelming them and possibilities for independent learning and self‐testing. Guided independent learning serves both the teacher and the student. Since the students are able to use the material outside of exercises, they can allocate the necessary time for comprehensive understanding through repetitive use of the annotations with questions and quizzes. The flexibility in time allocation has been shown as one of the great benefits of VM in respect of the learning outcomes (Husmann et al., 2009). Even though the digital platforms enable self‐directed learning the improved academic performance seems be related to the combination of traditional microscopy and lectures with the digital tools (Chimmalgi & Hortsch, 2022). The benefit of VM is not the final learning outcome but it can speed up the learning process (Helle et al., 2011). VM serves as a collaborative platform that ensures all students to have the same view of histology sections. It also provides a user‐friendly environment for asking questions, facilitating group work, and an enriched learning experience. During the COVID‐19 pandemic, QuPath Edu was used as a teaching tool in remote practical exercises. It was utilized both in the introduction for the whole group, as well the basis for discussion in the small groups in the remote sessions with success.

As the trend in medical education leans toward increasing numbers of students with fewer hours for teaching (Drake et al., 2009), VM is a useful tool to free up teachers' time as annotated material can be used as an introduction to the topic. The teacher is then able to concentrate more on the interaction with the students and questions arising during exercises. The students can return to the provided introduction easily during and after exercises. Comparable approaches to our slide tours have been executed on various platforms, ranging from conventional slide shows (Soma, 2016) to video introductions such as Shotgun Histology (Swerdlow, 2023). Guided introductions have been highly regarded as an effective and valuable learning method by residents and medical students during their hematopathology rotation (Soma, 2016). Here, Microsoft PowerPoint was employed as the platform for these introductions, but this choice came with certain challenges. Users occasionally found it challenging to follow the intended path, and large file sizes made sharing the slide show cumbersome. The advantage of a guided VM tour in QuPath Edu is that the slide tour is student paced and open for free exploration of the surrounding structures and zooming that are not possible using conventional slide shows or video material.

Web‐based VM platforms like our OpenMicroanatomy are also accessible on mobile devices, ensuring flexibility in learning. This allows students to engage with course materials anytime, anywhere, promoting continuous learning beyond the classroom. In basic education, the lightweight OpenMicroanatomy Web serves learning in many kinds of environments. This can bridge educational gaps and provide standardized resources globally. The utilization of QuPath Edu has been pilot‐tested in collaboration with Stellenbosch University in South Africa and University of Namibia in Namibia. The results of this pilot have demonstrated the feasibility of this platform for sharing teaching material between partners. The potential for collaboration between organizations is manifold. For instance, this can be achieved through the establishment of a common slide repository, which everyone can access, or by having multiple organizations on a single server. Each organization can upload their own slide collection to the slide repository and maintain ownership. In our approach, the slides in the repository can be utilized in multiple workspaces and in multiple lessons simultaneously. The lesson information, including annotations and slide tours, is stored in the project files in the lessons independent of the image files. This separation allows for the same slide to be utilized across various lessons simultaneously.

QuPath Edu and OpenMicroanatomy are by no means the only platform available for teaching histology. For example, HistoViewer (Sander & Golas, 2013), University of Leeds' Virtual Pathology Library (University of Leeds, 2024), Michigan Histology, and Virtual Microscopy Learning Resources (University of Michigan Medical School, 2024), PathScribe (Khvostikov et al., 2024) and several more (reviewed in Hassell et al. (2022)), are all resources which include a WSI viewer. QuPath Edu, however, in our opinion, combines all features of the previously mentioned solutions and exceeds them, and is the only one which is open source for others to contribute. Many of the existing VM resources referred above offer open educational material, but do not allow its modification. Here, we offer a tool that can be used for building customized material to be used locally or shared openly. The main barrier to QuPath Edu use is the technical knowledge needed to set up and maintain OpenMicroanatomy Server. After the initial investment the overhead cost of using it consists of the server costs, possible cloud storage costs and IT‐personnels time. The goal is to make setting up QuPath Edu and OpenMicroanatomy even more straightforward in the future. We plan to accept future open‐source contributions that can make QuPath Edu and OpenMicroanatomy even better and more accessible. What our platform currently lacks is a public slide collection. By building an open‐source community around QuPath Edu and OpenMicroanatomy, which includes a free slide collection, we will pursue more equal learning opportunities globally.

### 4.2 Scenarios for the future

In education, flexible evaluation tools are crucial. Currently, we use the Moodle environment for examinations, but QuPath Edu could be enhanced to include basic examination tools based on Edu's multiple‐choice or open‐ended question annotations, or more advanced features leveraging QuPath's capabilities. Because QuPath Edu is built on top of QuPath, in the future it could be possible to support elaborate exam scenarios using QuPath Edu with the help of personal projects. Students could, for example, be tasked with annotating specific types of cells or regions of tissue from a slide. Teachers would mark the correct cells or regions manually in advance or automate the task partially or entirely with the analysis tools of QuPath. Using QuPath scripts, it can then be automated to detect whether the student annotated the correct regions and identified them correctly. While this has yet to be implemented in practice, we have created a working proof‐of‐concept of this workflow. The QuPath Edu approach described here can equally well be used for medical and other students to learn about the histopathological changes of diseases along with the biological mechanisms of disease.

Recently, there has been discussion of the declining number of physician–researchers. Both intrinsic and extrinsic motivation are key factors in motivating medical students to become physician–researchers (Ommering et al., 2018). Especially the need for challenges was shown as a significant extrinsic factor. QuPath Edu is one way of introducing new challenges to medical students in the early stages of their studies. After using QuPath Edu for learning the basics of microanatomy, the student is already familiar with QuPath and its simple and intuitive user interface; this lowers the threshold for using it in research projects. Even though this alone does not create researchers, it can incorporate elements of medical research in the early curriculum and promote scientific thinking.

## 5 CONCLUSION

QuPath Edu and OpenMicroanatomy form a versatile free open‐source platform for basic microanatomy education and advanced image analysis training that respond and adapt to the educational needs arising from the development of digital tools in pathology research and clinical practice.

## AUTHOR CONTRIBUTIONS

**Aaron Yli‐Hallila:** software (lead), conceptualization (equal), investigation (lead), formal analysis (lead), visualization (lead), and writing original draft (lead). **Peter Bankhead:** software (supporting), funding acquisition (supporting), and reviewing and editing (equal). **Mark J. Arends:** reviewing and editing (equal). **Petri Lehenkari:** conceptualization (equal), funding acquisition (lead), supervision (equal), and reviewing and editing (equal). **Sanna Palosaari:** project administration (lead), conceptualization (equal), funding acquisition (lead), supervision (equal), and reviewing and editing (lead).

## FUNDING INFORMATION

This project was supported by Pathological Society of Great Britain and Ireland (Education grant EG 04211307) and Southern African and Finnish Higher Education Institutions' Network for Health and Well‐Being (SAFINET) funded by the Ministry for Education and Culture of Finland under its Global Internationalisation Programme 2021–2024.

## CONFLICT OF INTEREST STATEMENT

The authors declare no conflict of interest.

## Supporting information


Data S1.

## Data Availability

The data that support the findings of this study are available in the supplementary material of this article.
