# Supplementary material for: QuPath Edu and OpenMicroanatomy: Open‐source virtual microscopy tools for medical education
Source: J Anat. 2024 Nov 18;246(5):846–56. doi: 10.1111/joa.14172 (PMC11996698; doi:10.1111/joa.14172)
Supplement: Supplementary file 1 — Data S1. [file JOA-246-846-s001.docx]

**Supplementary file S1**

This supplementary file includes more in-depth information on how QuPath Edu and OpenMicroanatomy work from the technical perspective.

***Compatible file formats***

The OpenMicroanatomy slide repository supports scanners / file formats such as Aperio (.svs and .tif), DICOM (.dcm), Hamamatsu (.vms, .vmu, and .ndpi), Leica (.scn), MIRAX (.mrxs), Philips (.tiff), Sakura (.svslide), Trestle (.tif), Ventana (.bif and .tif), Zeiss (.czi), and generic tiled TIFF (.tif). Behind the scenes OpenMicroanatomy uses OpenSlide [1], which is an open-source tool for reading whole-slide images. OpenMicroanatomy can store the slides either locally on the server or on third-party cloud service such as Microsoft Azure [2], Amazon S3 [3], CSC Allas [4] and OMERO [5].

***OpenMicroanatomy Server***

A single OpenMicroanatomy Server can serve multiple organizations in parallel (see **Figure 1.**), which makes sharing of slides and materials easy. With OpenMicroanatomy’s permission and role system, access to slides and workspaces can easily be restricted, e.g only to users of the owner organization, to everyone, or to teachers only; see **Figure 1.** for an example setup. OpenMicroanatomy supports authenticating using either an email and password combination or with existing Microsoft 365 credentials by utilizing the Microsoft Identity Platform [6].


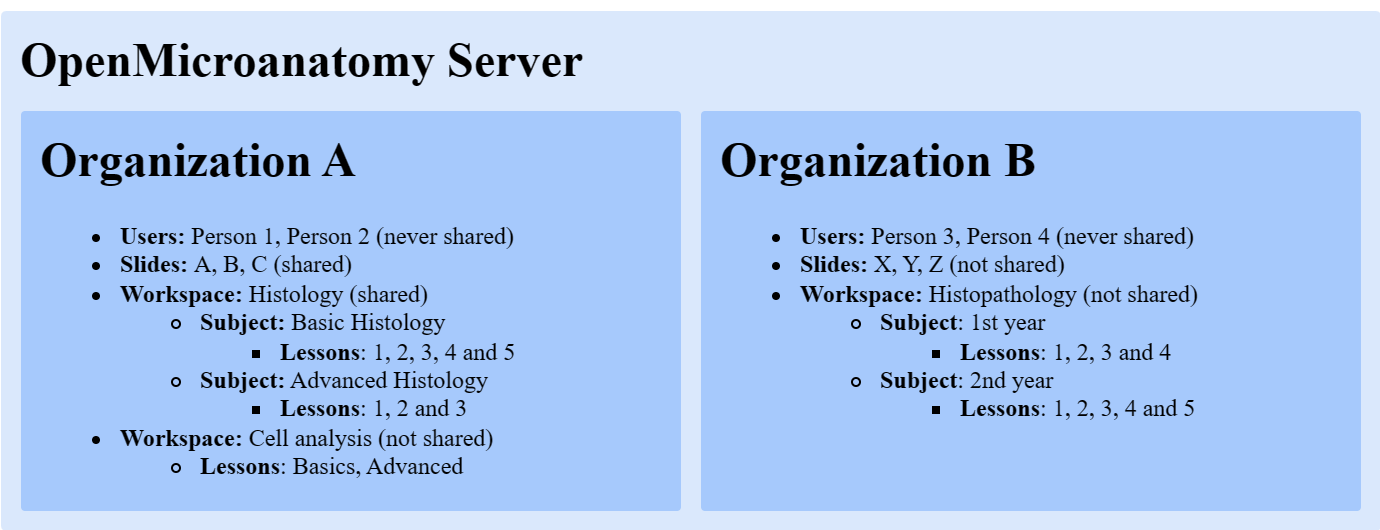


**Figure 1.** Example of an OpenMicroanatomy Server with two organizations (A and B). In this example all users regardless of organization can view the “Histology” workspace and the “Histopathology” workspace is available only to users of Organization B. Slides “A, B, C” are shared and can be used in lessons by teachers of both organizations. Slides “X, Y, Z” are exclusively available to teachers of Organization B.

To access slides remotely they need to be stored in a slide repository accessible via the internet. OMERO [5] is a free and open-source tool developed by the Open Microscopy Environment consortium, which provides a secure central repository for images. QuPath supports OMERO with the QuPath OMERO extension [7]. QuPath Edu and OpenMicroanatomy can be used with OMERO as the slide repository, but it also includes a built-in slide repository for a more streamlined process, as users then only need the QuPath Edu extension installed.

***Open-source technologies used***

QuPath Edu extension is written using the Java programming language, same as QuPath, utilizing third-party libraries such as Microsoft Authentication Library for Java [8]. OpenMicroanatomy Server is also written using Java, and includes third-party libraries such as Hibernate [9] for database management and Javalin [10] as the HTTP server, which is built on top of Jetty. OpenMicroanatomy Web is built using TypeScript and third-party libraries such as React [11] for the user-interface and OpenSeadragon [12] for the whole-slide image viewer.

**Supplementary file S2**

This supplementary file contains more in-depth information on the student survey.

The survey was used to assess usability of OpenMicroanatomy, how useful students perceived improved and new features of QuPath Edu and OpenMicroanatomy in learning histology, what type of annotations students preferred (explanations, questions, quizzes, or answers missing). All respondents gave informed consent for using their answers in this study. Demographic information such as gender, age range, degree program (physician or dentist) and previous university education were collected. The preferred way to access the materials was surveyed (OpenMicroanatomy, QuPath Edu, or no relevance).

For assessing usability the System Usability Scale (SUS) -questionnaire [13] and an unofficial Finnish translation [14] was used, which was slightly modified to reflect “application” instead of “system” (see **Table 1.**). The SUS-questionnaire is a simple 10-question form with five response options from Strongly agree to Strongly disagree. The questionnaire outputs a numeric value between 0-100, which can then be interpreted as the usability of the software in percentiles according to Sauro-Lewis grading scale [15].

Usefulness of the new features (descriptive annotations, annotations with multiple choice questions, annotations with question and answer, annotations with question and no answer, slide descriptions, and slide tours) were evaluated using a continuous ranging from -50 to 50 scale, where -50 = not useful, 0 = neutral and 50 = very useful.

| **Question: OpenMicroanatomy Web usability** | **Mean** | **Median** | **Standard deviation** |
| --- | --- | --- | --- |
| 1. I think that I would like to use this application frequently. | 4.32 | 4 | 0.70 |
| 2. I found the application unnecessarily complex. | 1.57 | 1 | 0.79 |
| 3. I thought the application was easy to use. | 4.40 | 5 | 0.77 |
| 4. I think that I would need the support of a technical person to be able to use this application. | 1.27 | 1 | 0.70 |
| 5. I found the various functions in this application were well integrated. | 4.09 | 4 | 0.79 |
| 6. I thought there was too much inconsistency in this application. | 1.59 | 1 | 0.77 |
| 7. I would imagine that most people would learn to use this application very quickly. | 4.55 | 5 | 0.76 |
| 8. I found the application very cumbersome to use. | 1.47 | 1 | 0.81 |
| 9. I felt very confident using the application. | 4.07 | 4 | 0.84 |
| 10. I needed to learn a lot of things before I could get going with this system. | 1.49 | 1 | 0.69 |

**Table 1**. Modified System Usability Scale (SUS) questionnaire used for OpenMicroanatomy Web and its results. An unofficial Finnish translation was used in the student survey, which had “system” changed into “application”. Values range from Strongly disagree (1), Disagree (2), Neutral (3), Agree (4) and Strongly agree (5).

# References

| [1] | A. Goode, B. Gilbert, J. Harkes, D. Jukic ja M. Satyanarayanan, ”OpenSlide: A vendor-neutral software foundation for digital pathology,” *Journal of pathology informatics,* osa/vuosik. 4, nro 27, 2013. |
| --- | --- |
| [2] | Microsoft, ”Cloud Computing Services \| Microsoft Azure,” 2023. [Online]. Available: https://azure.microsoft.com/en-us. [Haettu December 2023]. |
| [3] | Amazon, ”Cloud Object Storage – Amazon S3 – Amazon Web Services,” 2023. [Online]. Available: https://aws.amazon.com/s3/. [Haettu December 2023]. |
| [4] | CSC, ”Allas object storage - Docs CSC,” 2023. [Online]. Available: https://docs.csc.fi/data/Allas/. [Haettu December 2023]. |
| [5] | C. Allan, J.-M. Burel, J. Moore, C. Blackburn, M. Linkert, S. Loynton, D. Macdonald, W. J. Moore, C. Neves, A. Patterson, M. Porter, A. Tarkowska, B. Loranger, J. Avondo, I. Lagerstedt, L. Lianas, S. Leo, K. Hands, R. T. Hay, A. Patwardhan, C. Best, G. J. Kleywegt, G. Zanetti ja J. R. Swedlow, ”OMERO,” *Nature methods,* osa/vuosik. 9, nro 3, pp. 245-253, 2012. |
| [6] | Microsoft, ”Microsoft identity platform documentation,” 2023. [Online]. Available: https://learn.microsoft.com/en-us/entra/identity-platform/. [Haettu December 2023]. |
| [7] | QuPath Developers, ”QuPath OMERO extension,” 2023. [Online]. Available: https://github.com/qupath/qupath-extension-omero. [Haettu December 2023]. |
| [8] | Microsoft, ”Microsoft Authentication Library for Java,” 2023. [Online]. Available: https://learn.microsoft.com/en-us/entra/msal/java/. [Haettu December 2023]. |
| [9] | Hibernate Developers, ”https://hibernate.org/,” 2023. [Online]. [Haettu December 2023]. |
| [10] | Javalin Developers, ”https://javalin.io/,” 2023. [Online]. [Haettu December 2023]. |
| [11] | Meta Open Source, ”https://react.dev/,” 2023. [Online]. [Haettu December 2023]. |
| [12] | OpenSeadragon Developers, ”https://openseadragon.github.io/,” 2023. [Online]. [Haettu December 2023]. |
| [13] | J. Brooke, ”SUS: A quick and dirty usability scale,” *Usability Evaluation in Industry,* osa/vuosik. 189, 11 1995. |
| [14] | T. Jokela, ”SUS (System Usability Scale) suomeksi,” 2013. [Online]. Available: http://kaytettavyysnavigoija.blogspot.com/2018/08/sus-system-usability-scale-suomeksi.html. [Haettu December 2023]. |
| [15] | J. Sauro ja J. R. Lewis, Quantifying the User Experience: Practical Statistics for User Research, 2nd toim., Cambridge: Elsevier, 2016. |
